# Return to Learn: Preferences of College Educators When Receiving Concussion Medical Notes

**DOI:** 10.1089/neur.2022.0012

**Published:** 2022-04-25

**Authors:** Zachary W. Bevilacqua, Donetta Cothran, Devin Rettke, David Koceja, Thomas Nelson-Laird, Keisuke Kawata

**Affiliations:** ^1^ Department of Exercise Science, Wegmans School of Health and Nutrition, Rochester Institute of Technology, Rochester, New York, USA.; ^2^ Department of Kinesiology, School of Public Health, School of Education, Indiana University Bloomington, Bloomington, Indiana, USA.; ^3^ Department of Postsecondary Research, School of Education, Indiana University Bloomington, Bloomington, Indiana, USA.

**Keywords:** college, concussion, medical notes, return to learn, return to school

## Abstract

The aim of this work is to uncover the preferences and perspectives of college educators as they interpret medical documentation outlining medically requested return-to-learn (RTL) instructions. Participants were recruited from five colleges across campus at a large Midwest public university. They each engaged in a private, one-on-one, audio-recorded interview. All recordings were transcribed and inductively analyzed using a grounded theory approach and two-coder system. All codes and themes were finalized once agreement was reached by both coders. Resultant themes from axial coding had to represent the voices of at least 80% of participants. Three characteristics emerged as being desired by college educators: brevity, clarity, and direction. Educators also expressed considerably less utility with medical documentation designed for pediatric students with concussion. College educators desire medical notes that are brief, clear, and provide straightforward direction, in addition to documentation that is tailored for the college setting.

## Introduction

Pediatric return-to-learn (RTL) literature has identified the importance of providing medical documentation to students with concussion, highlighting the increased likelihood of receiving academic accommodations when a note is present.^1–3^ Grubenhoff and colleagues, however, showed that 96% of sampled students within a K-12 cohort received their school accommodations informally, hinting that these settings tend to support acutely concussed students with academic adjustments, over methods that require formal medical documentation (e.g., 504 Plan, Individualized Education Program).^4^ Others corroborate this by showing how 70% of high school principals would provide classroom adjustments to a student without a medical provider's note.^5^ RTL commentary further acknowledges the similarity between normal recovery time from adolescent concussion (i.e., 3–4 weeks) and when formal accommodations (504 Plan) would be appropriate to implement, profiling the necessity of formal accommodations as uncommon.^6^ Acknowledging the tendency toward informal delivery of academic supports, we can begin to explain why systematic review of K-12 RTL found no articles discussing the beneficial relationship between 504 accommodations and a faster RTL.^7^

Still in its beginnings, the delivery of academic support within the college setting is concurrently being explored. Recent grounded theory data indicate that awarding academic accommodations in the college environment largely hinges upon the presence of a medical note.^8^ College educators also identified medical documentation as a necessary tool to effectively legitimize a student's concussion, predicating their reliance upon medical expertise.^8^ These data suggest that medical notes in the college setting act as prerequisites to initiating classroom support, versus K-12, which appear to favor informal adjustments to the classroom environment. These divergent characteristics prompted this group to investigate the utility of medical notes used to support students as they RTL. Thus, the primary aim of this investigation was to uncover educator preferences as they evaluate the usefulness of medical notes containing RTL instructions.

## Methods

### Participants

Twenty-three college educators from a large public institution were recruited from five schools across campus (Public Health, Business, Education, Optometry, and Public and Environmental Health). All participants were current faculty with teaching responsibilities and a previous history of instructing students with concussion. Permission to interview participants was given by the Indiana University Institutional Review Board and received exempt status.

### Interview

Semistructured, private, audio-recorded, one-on-one interviews were utilized. Interviews were recorded by voice recorder in a closed-door location of the participant's choosing. All interviews were conducted by a single researcher and were an average of 62 min in length.

### Interview materials: medical notes

During the interview, participants were asked to review two sample medical notes (Supplementary Appendix SA; Supplementary Appendix SB). Supplementary Appendix SA was an RTL document published for K-12 students,^1^ whereas Supplementary Appendix SB was an RTL note currently in use at the studied university. Participants were blinded to the origin of these documents. Participants reviewed the K-12 note first and were given the following instructions: “Here is an example of a medical note for a student recovering from concussion. Read over it and tell me what you like, don't like, find helpful, and then we'll discuss your impressions.”

After giving their opinions, participants were then given the college note to repeat the process. Paper versions of these notes were used, allowing participants to physically annotate as they reviewed.

### Data analysis 

Transcripts were independently open- and axially coded by two researchers. Coding was completed using Microsoft Word^®^ (Microsoft Corporation, Redmond, WA), which included an iterative process of grouping segments of text that embodied a similar meaning. From this, desirable medical note characteristics were identified. In order for a characteristic to be desirable and representative of the sample, it had to include matching codes from at least 80% of the sample. This cut-off value was chosen because it signifies robust homogeneity among educators, while not excluding characteristics that did not reach unanimous representation.

### Trustworthiness measures

Credibility methods (triangulation, member checks, peer debriefing, and two-coders) and confirmability methods (audit trail, journaling) were incorporated to maintain methodological rigor and mitigate researcher bias.^9^

## Results

### Demographics

Twenty-three faculty volunteered to participate in the investigation. Table 1 illustrates their demographics.

**Table 1.** Demographics

School	Sex	Age (range)	Years teaching in college (range)	Academic rank represented: NTT, TT	No. of instructed concussed students (range)	Class sizes (range)
Public Health	F = 9	30–69	6–40	NTT; TT	1–7	1–250
	M = 6	30–69	8–31	NTT; TT	1–10	3–150
Business	F = 1	50–59	10	NTT	18	24–80
	M = 4	30–79	6–45	NTT; TT	2–10	15–275
Education	F = 1	70–79	40	TT	2	5–24
Optometry	M = 1	50–59	14	TT	2	10–80
Public and Environmental Health	M = 1	40–49	15	TT	2	8–100

NTT, non-tenure-track; TT, tenure-track.

### Themes

Data analysis revealed three themes that characterized a useful note; 1) brevity, 2) clarity, and 3) direction. Here we will provide participant responses that delineate the source of these characteristics.

### Brevity

The cohort habitually commented on the length and density of information within the K-12 note (Supplementary Appendix SA): “It's too long. Way, way too long.”; and “I would never look at this whole thing. I would just ask the student, what can you do? … You tell me what you're ready to do, when you're ready to do it.”

Some content even appeared nonessential, as summarized here:

“On the surface, the difference between these two notes may be a collegiate level for Appendix B, and high school or middle school for Appendix A. I almost think that the different level of the student would tailor into how these notes are received. Cause again, for college students, the instructor is only going to see them a couple times a week, so it's (the note) only pertaining to their individual class. So I think that a collegiate instructor just needs to know that this (concussion) is a condition, this is why you cannot come to class, do your assignments, take your exams, this is when you can, and this is what to expect from me. Where I think, the high school or middle school teacher who technically will be in proximity to a student throughout the day may need to be more in tune with the signs and symptoms, how students are feeling, how they may react, what they can and cannot do throughout the day, and what things may help and hinder them. So I think the symptoms checklist and the personal narrative of Appendix A would be more beneficial for younger students and teachers that have proximity with them all day, where the official letter type of format of Appendix B would be better for a collegiate instructor to legitimize the short period of time they have them throughout the week.”

Contrastingly, two participants with a background in K-12 pedagogy found value in the list of accommodations outlined in the K-12 note: “I like understanding that there is a range of symptoms. I like that it breaks it down and it's informative, rather than just saying, ‘don't do this, don't do that,’ and these are some of the symptoms.”; and “We're going to get folks who won't read this. But, for those who actually read it, I think this is amazing.”

### Clarity

Because participants were given paper copies of both notes, we were able to identify words and phrases that were routinely marked as elucidating or helpful. According to the K-12 note: “many students will benefit from some accommodations”; and “Recovery typically takes between several days to several weeks.” According to the college note: “mild traumatic brain injury”; “students who appear healthy may actually be ill”; “the student is responsible for following up with you”; “no class attendance, homework, exams or screen time for 6 days”; and “the student will be returning to the Health Center on xx/xx/xx.”

### Direction

Participants identified the goal of a medical note to be an “action item,” requesting the educator to perform a duty or provide support for the student. Therefore, the following responses iterate the ease-of-use in finding the action item(s) within a note.

“The format of Appendix B is useful for doing what I want to do… So when I got the email (note), I remembered scanning it for whoever has a concussion. Okay, she has a concussion. And then I went like this, what do I have to do? What do I have to do? Which is this bottom part (of Appendix B).”

“Okay, I like this one (Appendix B). I like it a lot better. I mean it says they're going back to the health center. It says no class attendance, homework exams or screen time for six days, then to attend class, to return to completing homework assignments, which I would accommodate … it also indicates they're going to go in for a recheck, so then it's not up to me to evaluate their current situation.”

Although informational, educators found the K-12 note to be non-specific and left many unsure as to what was being requested.

“It said the student can return to school when they can concentrate on schoolwork for 30 minutes before symptoms worsened significantly. Okay, so let's say they return to school, but they're in my lecture hall and in 15 minutes they can't tolerate it, and they leave the classroom. Does that mean I have to pull them out as an instructor? Do they go back to the physician? What does that mean for me?”

“There's no to-do here. It says, here's the symptom, here's the effect on school learning, here's an accommodation that I assume is helpful, and I could link it back to the current symptoms, but what do I do with this information is the question I have. I understand the symptom. I understand the effect on learning. I understand what accommodation is helpful. Is the goal my understanding, or am I supposed to do something about it?”

## Discussion

The presented data introduce the preferences of college educators as they examine medical notes containing RTL instructions. Brevity, clarity, and direction emerged as sought-after characteristics. The data also highlight specific phrases that offered clarity and straightforward guidance. Overall, the current findings lead us to hypothesize that college educators seek medical documentation with the capacity to quickly answer the following questions: 1) “Who is involved (student, campus entity)?”; 2) “What is the illness/injury?”; and 3) “What must I do as an instructor, and for how long?”.

One participant detailed at length how they would receive the given medical notes, placing a specific emphasis on why the accommodations table in Supplementary Appendix SA holds greater relevance to K-12 educators who share a physical proximity to students throughout the day. This perspective of providing K-12 educators with an “accommodation handbook” appears to have been previously presented by Halstead and colleagues, who endorsed the need to “educate general education teachers about concussion, specifically on how to make short-term academic adjustments in the general education classroom.”^6^ Additional investigations corroborate the importance of providing educational materials for K-12 teachers.^10–12^ These data, however, contrast the sentiments shared by our cohort, who stressed the importance of clear direction from medical documentation versus the freedom of using their understanding of concussion to subjectively assess and implement academic adjustments. Other qualitative research echoes this, identifying how middle and high school teachers “expressed the need for specific guidance on how to support their students with concussion.”^13^ Further, providing instructional programs for educators with the intent to equip them in the future departs from the role that educators have assumed within the RTL team. College educators have identified their role as “peripheral and responsive,” with K-12 educators portraying their duty as “reactive, not preventive, when it comes to concussion.”^8,13^ Taken together, these data propose that K-12 and college educators seek a level guidance that is contrary to what is presumed in the literature.

Desiring objective direction from qualified medical personnel is logical, especially given that previous work has shown that completion of a concussion education course by educators inadequately translates to classroom application 6 months later.^14^ Further, giving teachers the autonomy to implement classroom supports for a student with concussion is conceivably negligent, given that this effectively gives educators the authority to dictate how a temporary neurological disability is academically treated—a duty for qualified medical personnel.

In keeping with the characteristics elected by our sample, this group proposes that the following note (Supplementary Appendix SC) be used as a starting point toward the development of an efficacious medical document, used to convey RTL guidance to college educators. We should note, however, that the logistical and legal discrepancies between K-12 and college settings warrant this note to be retailored if extrapolated to the K-12 setting.

### Limitations

The current study is not without limitations. First, our cohort was asked to analyze a sample medical note that was currently in use at the studied university. Additionally, inclusion criteria dictated that all participants have previously instructed a student recovering from concussion. Therefore, study participants may have already been familiar with Supplementary Appendix SB, and or formulated opinions of its utility, inserting bias. Second, the current findings represent the voice of a large public university, which may differ from other universities. Last, despite reaching data saturation, 23 participants is not a sufficient sample to represent the full landscape of higher education, nor does it speak for educators who have not encountered a student recovering from concussion.

## Conclusion

The sampled cohort of college educators identified three preferential characteristics to include in RTL notes: 1) brevity, 2) clarity, and 3) direction. Educators also expressed a preference toward the content and length of the sample college note, indicating that documentation for RTL should be tailored to the level of academia in which it will be implemented. As efforts continue to mold RTL into a robust prong of post-concussion management, identifying setting-specific distinctions becomes prudent. Future investigations should use these findings to determine the utility of other notes in circulation, in addition to our proposed note for higher education. Widespread perceptions of this novel document may also act as a proxy for the accuracy of our identified themes. Finally, follow-up studies must include educators with no RTL experience, to address whether familiarity dictates a person's RTL viewpoint.

## Supplementary Material

Supplemental data

Supplemental data

Supplemental data

## Supplementary Material

Supplementary Appendix SA

Supplementary Appendix SB

Supplementary Appendix SC

## Abbreviation Used

RTL: return to learn
