# Supplementary material for: Return to Learn: Preferences of College Educators When Receiving Concussion Medical Notes
Source: Neurotrauma Rep. 2022 Apr 25;3(1):185–9. doi: 10.1089/neur.2022.0012 (PMC9080999; doi:10.1089/neur.2022.0012)
Supplement: Supplemental data [file Supp_AppSA.pdf]

APPENDIX A

POST-CONCUSSION RETURN TO SCHOOL LETTER

Dear School Staff:

[Student]__________________________ sustained a concussion on ___[Date]___________.

Recovery typically takes between several days to several weeks. The student should return to school as soon as they can tolerate it but many students will benefit from some accommodations to their school programme as they recover. As symptoms resolve and the student's learning/cognitive functioning returns to normal, s/he can gradually progress to their normal school day with reduced supports.

Current Symptoms: The student is currently reporting the following symptoms as indicated by the (√) below. These can be viewed as targets for supportive classroom accommodations to assist a successful return. See suggested supports for these symptoms on page 2.

PHYSICAL		COGNITIVE	EMOTIONAL
□ Headaches	□ Fatigue	□ Feeling mentally foggy	□ Irritability
□ Sensitivity to light	□ Sensitivity to noise	□ Memory problems	□ Anxiety/ nervousness
□ Blurry/double vision	□ Nausea/ vomiting	□ Slowed thinking/ performance	□ Sadness
□ Balance Problems	□ Dizziness	□ Difficulty concentrating	□ Feeling more emotional

Return to School: The student can return to school when:

(1) S/he can concentrate on school work for 30 minutes before symptoms worsen significantly.
(2) Symptom exacerbation reduces/resolves with cognitive rest breaks, allowing return to activity.

Based on the current symptoms, he/she is ___ *permitted to return to school.*
___ *is excused for* _____ *days*

Safety Restrictions: To reduce risk for re-injury, there should be

- ■ No physical (risk) activity during recess
- ■ No Physical Education (Gym) class
- ■ No sports participation
- ■ Other:___________________________

Physical Activity: Mild-moderate symptom-limited exercise (walking) daily is permitted.

Health Care Provider Signature ____________________________ Date_____________________

Contact Information____

Provision of School Supports: Listed are some suggested accommodations for consideration by school personnel, tailored to the student's specific symptoms. Certain subjects, such as math, reading/language, arts, science and social studies, may pose greater problems for students returning after a concussion and may require more accommodations.

Post-concussion symptom	Effect on school learning	Accommodation
Physical Symptoms		
Headache	Difficulty concentrating	Frequent breaks, quiet area, hydration
Fatigue	Decreased attention, concentration, low energy	Frequent breaks, shortened day, attendance in fewer classes
Light/noise sensitivity	Worsening symptoms (headache)	Sunglasses, ear plugs/headphones, avoid noisy areas (cafeterias, assemblies, sport events, music class), limit computer work
Dizziness/ balance	Unsteadiness when walking, room feels like it is spinning	Elevator/lift pass (if available)<br>Class transition before bell
Cognitive Symptoms		
Difficulty concentrating	Limited focus on schoolwork	Shorter assignments, decreased workload, frequent breaks, having someone read out loud, more time to complete assignments/tests, quiet area to complete work
Working/ short-term memory	Forgetting instructions, oral lecture, reading material, thoughts during tasks	Repetition; Written instructions<br>Provide student with teacher generated class notes
Difficulty remembering	Difficulty retaining new information, remembering instructions, accessing learned information	Written instructions, smaller amounts to learn, repetition
Slow speed of performance / process	Unable to keep pace with work load, slower reading/ writing/calculation<br>Difficulty processing verbal information effectively	Extended time to complete coursework, assignments, tests<br>Reduce/slow down verbal information and check for comprehension
Emotional Symptoms		
Anxiety	Decreased attention or concentration, overexertion to avoid falling behind	Reassurance and support from teachers about accommodations, reduced workload
Irritability	Poor tolerance for stress (social, academic load)	Reduce stimulation and stressors (e.g., overwhelmed with missing work)

Note: Further information on the Symptom-Targeted Academic Management Plan (STAMP) can be found in Gioia, GA. Operationalizing Active Collaborative Management of Mild TBI in the School Setting. In: I. Gagnon & A. Ptito eds. Sports Concussions: Recovery and Management. Boca Raton, FL: CRC Press; in press.
