# Supplementary material for: Return to Learn: Preferences of College Educators When Receiving Concussion Medical Notes
Source: Neurotrauma Rep. 2022 Apr 25;3(1):185–9. doi: 10.1089/neur.2022.0012 (PMC9080999; doi:10.1089/neur.2022.0012)
Supplement: Supplemental data [file Supp_AppSB.docx]

**SUPPLEMENTARY APPENDIX B**

Dear Education Specialist,

**The student copied above** is in your class and is working with our office in conjunction with the XX Health Center. In collaboration with the Health Center, we developed a protocol for students who are recovering from a mild traumatic brain injury as their condition progresses from full mental rest to return to full activity. This is considered a temporary impairment and not covered under ADA.

We recognize that students who return to a full cognitive workload too soon after sustaining a concussion may experience worsening symptoms or a delay in full recovery. Returning to school after a concussion is a very individualized process. Class attendance may be limited. Students often have light and noise sensitivity, headache, and trouble focusing, concentrating and remembering. Your cooperation with the medical recommendations below often helps to lessen the symptoms and may allow the student to return sooner to full participation. It is important to keep in mind that students who appear healthy may actually be ill.

Each of the stages outlined below are part of a “Return to Learn” progression, and should take place when symptoms are tolerable and manageable and as directed by a health care provider. Please visit www.comIU Health Center website for more information about concussions.

It is up to each instructor to decide whether to excuse an absence/assignments/exams and if any alternative arrangements will be required. The final disposition of accommodations depends on an agreement made between the student and the instructor. The student is responsible for following up with you regarding specific arrangements in your class.

**After an injury sustained on 10/XX/19 and visiting the XX Health Center 10/XX/19** the student brings a recommendation to our office that indicates their current level of functioning.  The physician has recommended:

- **Stage 1: Complete Cognitive Rest:**
  - **No class attendance, homework, exams or screen time for 6 days.**
  - **Attend classes**
  - **Return to completing homework assignments, including complex assignments.**
  - **No exams.**

Updates will be sent following each appointment with the XX Health Center.  The student will be returning to the Health Center on **10/XX/19**
