# Supplementary material for: Return to Learn: Preferences of College Educators When Receiving Concussion Medical Notes
Source: Neurotrauma Rep. 2022 Apr 25;3(1):185–9. doi: 10.1089/neur.2022.0012 (PMC9080999; doi:10.1089/neur.2022.0012)
Supplement: Supplemental data [file Supp_AppSC.docx]

**SUPPLEMENTARY APPENDIX C**

(Insert University Letterhead)

Dear Professor,

The Disability Services Office is reaching out to notify you that your student, **(*student’s name*), was diagnosed with a mild traumatic brain injury, and has been granted the following classroom accommodations until DD/MM/YY:**

- *(Accommodation)*
- *(Accommodation)*
- *(Accommodation)*

**What is being asked of you:**

Allow the above accommodations until DD/MM/YY.

**Keep in mind:**

- A mild traumatic brain injury, otherwise known as **concussion, is a temporary disability protected by the Americans with Disabilities Act.^1^**
- The student is working with the (*campus entities, if applicable, e.g. Health Center*) and the Disability Services Office to ensure a prompt recovery.
- Students who look fine may actually be very ill.
- Concussion is an individualized disability, requiring several days to several weeks to heal, and often involves “invisible” symptoms (e.g. headache, anxiety, light sensitivity) that are not outwardly apparent.

**Moving forward:**

**(*student’s name*) will return to our office on DD/MM/YY. Subsequent directions will be electronically sent to you by our office after that visit; however, it is the student’s responsibility to keep you updated as to their progress.**

*Please contact us with any question: (Insert disability office representative’s contact information – name, position or title, physical office location, email address, phone number, fax number, disability office’s website URL)*

1. Division USD of JCRi: Final rule implementing the ADA amendments act of 2008. (2016). Accessed: August 11, 2021: http://www.ada.gov/regs2016/final_rule_adaaa.html.
